# Pulp and paper mill wastes: utilizations and prospects for high value-added biomaterials

**DOI:** 10.1186/s40643-021-00385-3

**Published:** 2021-04-29

**Authors:** Adane Haile, Gemeda Gebino Gelebo, Tamrat Tesfaye, Wassie Mengie, Million Ayele Mebrate, Amare Abuhay, Derseh Yilie Limeneh

**Affiliations:** Biorefinery Research Centre, Ethiopian Institute of Textile and Fashion Technology, Bahir Dar University, Bahir Dar, Ethiopia

**Keywords:** Paper mill, Biorefinery, Pulping waste, Biomass, Biomaterials, High value-added

## Abstract

A wide variety of biomass is available all around the world. Most of the biomass exists as a by-product from manufacturing industries. Pulp and paper mills contribute to a higher amount of these biomasses mostly discarded in the landfills creating an environmental burden. Biomasses from other sources have been used to produce different kinds and grades of biomaterials such as those used in industrial and medical applications. The present review aims to investigate the availability of biomass from pulp and paper mills and show sustainable routes for the production of high value-added biomaterials. The study reveals that using conventional and integrated biorefinery technology the ample variety and quantity of waste generated from pulp and paper mills can be converted into wealth. As per the findings of the current review, it is shown that high-performance carbon fiber and bioplastic can be manufactured from black liquor of pulping waste; the cellulosic waste from sawdust and sludge can be utilized for the synthesis of CNC and regenerated fibers such as viscose rayon and acetate; the mineral-based pulping wastes and fly ash can be used for manufacturing of different kinds of biocomposites. The different biomaterials obtained from the pulp and paper mill biomass can be used for versatile applications including conventional, high performance, and smart materials. Through customization and optimization of the conversion techniques and product manufacturing schemes, a variety of engineering materials can be obtained from pulp and paper mill wastes realizing the current global waste to wealth developmental approach.

## Introduction

The demand and use of pulp and paper have marked the levels of civilization and development of many societies (Armstrong et al. 1998; Lwako et al. 2013). The pulp and paper mills mainly utilize wood sources for the production of pulp and paper. Though appropriate quantitative analysis of waste produced from pulp and paper mills has not been done yet; in general only a few percent of the wood sources are utilized for the actual pulp and paper production (Bajpai 2015b). The rest is discarded as solid and liquid waste. Considering the developing countries' scenario both quantitative and qualitative analysis of biomass generated from the mills has not been done yet. Except for a few percent of wastepaper utilized in paper recycling the rest of the waste is left in the landfill or incinerated with no commercial significance; besides no satisfactory waste recycling scheme is implemented. Furthermore, in developing countries, the trend towards the conversion of the wastes into valuable materials is not practiced appreciably (Bajpai 2015d).

Environmental sustainability is a priority issue as far as sustainable development of nations is progressive. It is also a global concern recently to get rid of the factors that counteract environmental sustainability such as climate change, natural sources depletion, ecosystem prevention, and environmental degradation (McKinnon 2010; Bajpai 2018b; Carroll and Turpin 2002). The wastes from pulp mills one way or the other have a contributory effect on these factors and a feasible remedy needs to be designed for the overall protection of the environment for its sustainability. The pulp industry wastewater is generated from several sources like washing of raw wood materials before pulping, washing of cooked pulp and bleaching pulp, and finally, chemical recovery system (Song et al. 2019). Solid waste is produced primarily from the rejection of screening, primary and secondary sludge from wastewater management, and lime sludge from the chemical recovery system (Đurđević et al. 2020). Currently, a large amount of solid and liquid wastes are generated by the pulp and paper mills and environmental aspects are becoming a major concern that needs intervention.

The waste generated from pulp and paper mills adversely affects the environment from different perspectives. The emissions from pulp and paper industries have a significant effect on the environment. The generated waste from the pulp and paper industry causes severe harm to aquatic life, disturbs the food chain, and also causes various health implications (Gupta et al. 2019). The effect of pollutants on the environment has been assessed many times, but their mitigation still has a significant challenge (Gupta et al. 2019). Besides, many of the developing countries do not produce pulp. These paper mills use imported pulp and waste paper as raw materials.

It is vital from an environmental and socio-economic social point of view to utilize appropriate technology to convert the wastes from pulp and paper mills to highly valuable products that will have an impact on everyday life. This requires a thorough analysis of generated biomass quantitatively and qualitatively and the current review is limited to envisaging the availability of biomass from the said mills, the current utilization scheme, and the potentiality for biomaterial production and diversified application. Besides protection of the environment from undesired waste impacts, utilization of the wastes will greatly support the product diversity scheme and economic growth potency (Landrigan and Fuller 2015).

### Overview of pulp and paper industry

#### Status of pulp and paper industry at a global perspective

**Fig. 1:**
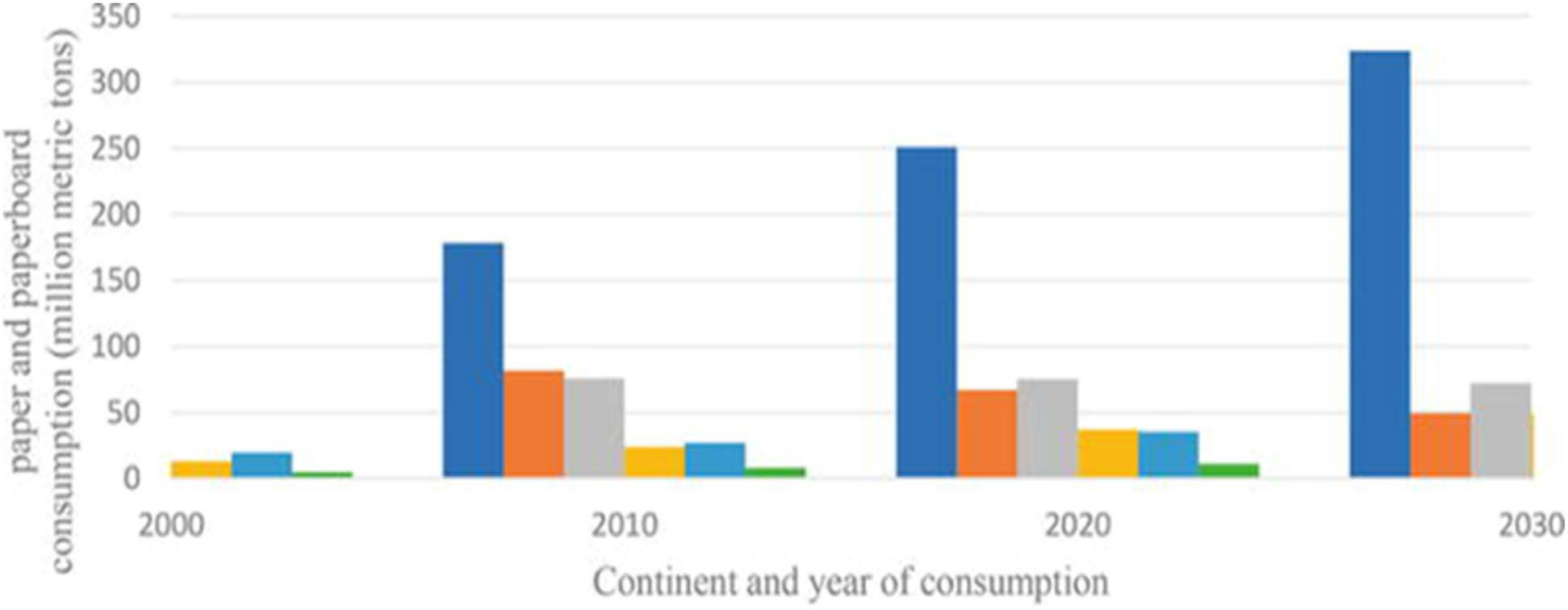
Yearly trend and future forecast on world paper production

As one of the most important industries worldwide pulp and paper mills supply varied products for around 5 billion people for versatile applications (Bajpai, 2015c). The paper is manufactured from pulp and a huge amount of trees is consumed for the pulp production with different shares by different countries in the world (Brännvall 2009, Cabreha 2017). The overall trend in the consumption of paper and paperboard by different countries and the forecasted consumption is shown in Fig. 1.

**Fig. 2:**
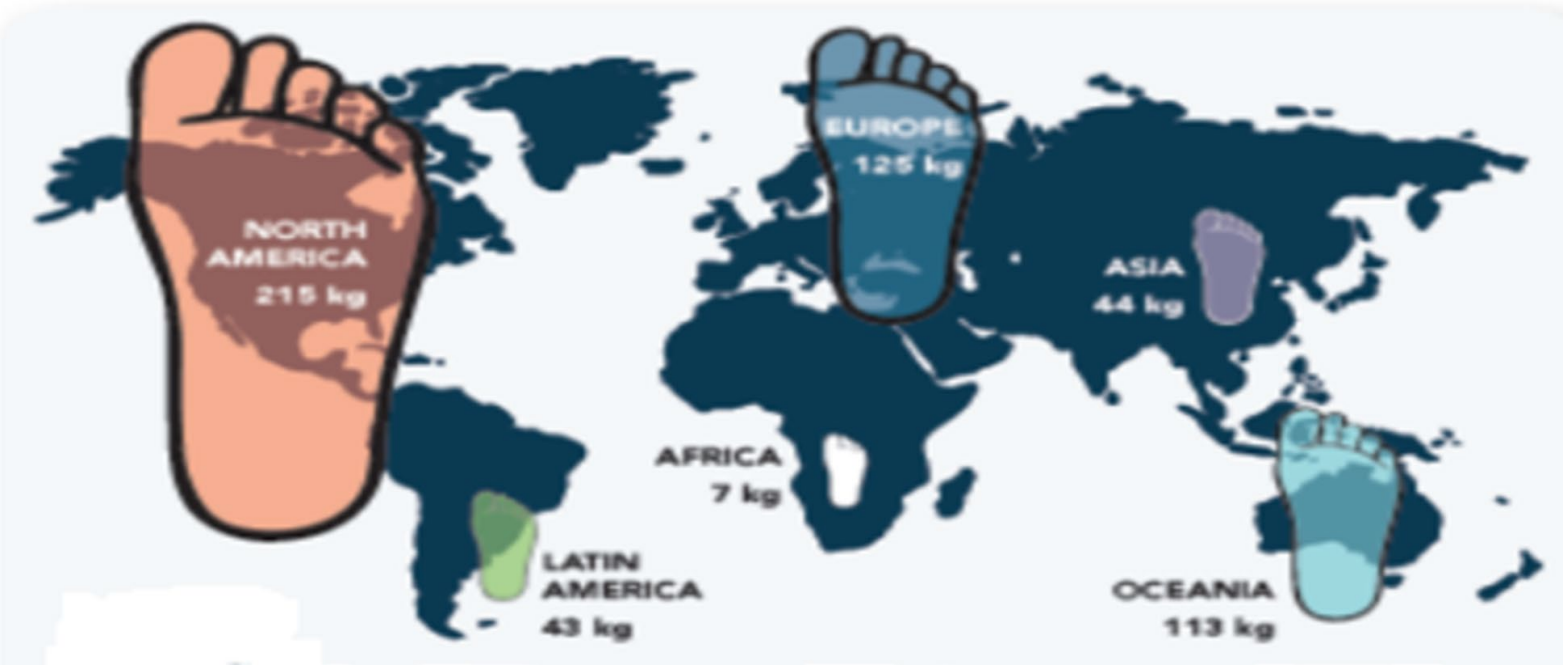
Global consumption of paper by major countries

The pulp and paper industry is one of the largest industries in the world. The consumption of paper is dominated by North American, Northern European, and East Asian countries (Fig. 2) (Diesen et al. 1998; Gopal et al. 2019; Hammett et al. 2001). Other dominant countries with significant pulp and paper industries are Australia and South America. A massive and key contribution is expected from China and India in the upcoming few years.

Considering the significant variation in consumption of paper products per person from the country to country the global consumption of paper and paper board is estimated on average around 55 kg/person per year. The utilization of paper and board is increasing year to year though digitalization is expected to influence production and consumption in different ways. The dominant countries like North America, Europe, and Asia and low producers and consumers like African countries are still utilizing huge amounts of paper.

The key components in the paper are cellulose sheets. There are also other constituents derived from the woody and related raw materials. The raw materials for manufacturing pulp and paper are ample and mainly consist of cellulose. Besides wood which is a chief source of cellulose, recycled paper and other cellulose bearing agricultural residues can be used as a raw material. The scenario in developing countries showed around 60% cellulose is obtained from jute, sisal, bamboo, bagasse, and similar non-wood resources.

Wood is considered the primary raw material and the major source of pulp in paper production (Eugenio et al. 2019). The gross characteristic composition of wood indicates its major constituents are cellulose, lignin, and hemicellulose. It also contains a trace amount of extractives of different types.

Numerical observation revealed that wood contains around 40–50% of cellulose (Table 1) (Sjöström and Westermark 1999). Considering ultimate properties such as strength and printability of paper it is possible to use hardwood and softwoods separately or combined in the manufacturing process. (Liu et al. 2018; Asmare 2017).

**Table 1:** Composition of wood

S. N	Component	% Composition
1	Cellulose	40–50%
2	Hemicellulose	25–30
3	Lignin	25–30
4	Extractives	Minor

An onsite availability study has been conducted on paper mills in Ethiopia. According to the study report, 25 paper enterprises are assessed and of which 12 companies are effective in producing different kinds of paper products with measurable production losses (Table 2).

**Table 2:** Status of paper manufacturing in Ethiopia

Company Name	Planed production (tonnes per year)	Production loss/year (%)	Product type
Ethiopia Pulp and Paper S.C	6000	5–15	Printing and Writing Paper, Stationery Paper, Paper board, Corrugated box, Wood free and manila papers, Kraft liners, Test liners, Fluting papers
Anmol Products Ethiopia	12,000–14,000	6.5–10.8	Printing writing papers, Wood free and manila papers, Kraft liners, Test liners, Fluting papers
Yekatit Paper Converting	1400	5–10	Different paper products
DA Packaging	2000	5–10	Kraft liners, Test liners, Fluting papers, Corrugated cartons
Barguba Trading	8000	7.2–13.5	Different paper products
Mamco Paper Products	200	2–10	Paper and tissue products
Burayu Development	10,000–11,500	15–18.5	Carton boxes, Trays, Packets
Minaye Packaging	3500	2.8–12.3	Carton
Balaje Packaging	1800	3–11.2	Carton
Unlimited Packaging	20,000	4.2–15	Carton
Seven Hills Packaging	7000	2–7.6	Carton
Akless Paper Product	350	5–8.6	Different Paper products

The main aim of the preliminary study was to determine the availability of waste and possible ingredients for biomaterial synthesis. The production loss indicated the potential availability of wastes and allied potential ingredients from the mills. The availability study in the specified country ensured a potential for conversion of pulp and paper mill biomass into biomaterials. Limitation to produce pulp indigenously can also be solved for the satisfaction of the demand for paper and paper products.

#### Overview on pulp and paper manufacturing process

The availability of raw materials for pulp and paper manufacturing is versatile and mainly cellulosic fibers from wood and selected plants are utilized. Besides wood used most widely in many countries of the world plant resources such as sugar cane residues, residues on cottonseed, and remnants from flax and rags are also used (Osman Khider et al. 2012; Asmare 2017; Enayati et al. 2009; Jahan et al. 2004; Tutus et al. 2010). Besides, in case of scarcity of virgin raw material resources recycled paper after proper after-treatment, through appropriate blending and recycling process, is used in some paper manufacturing trends.

**Fig. 3:**
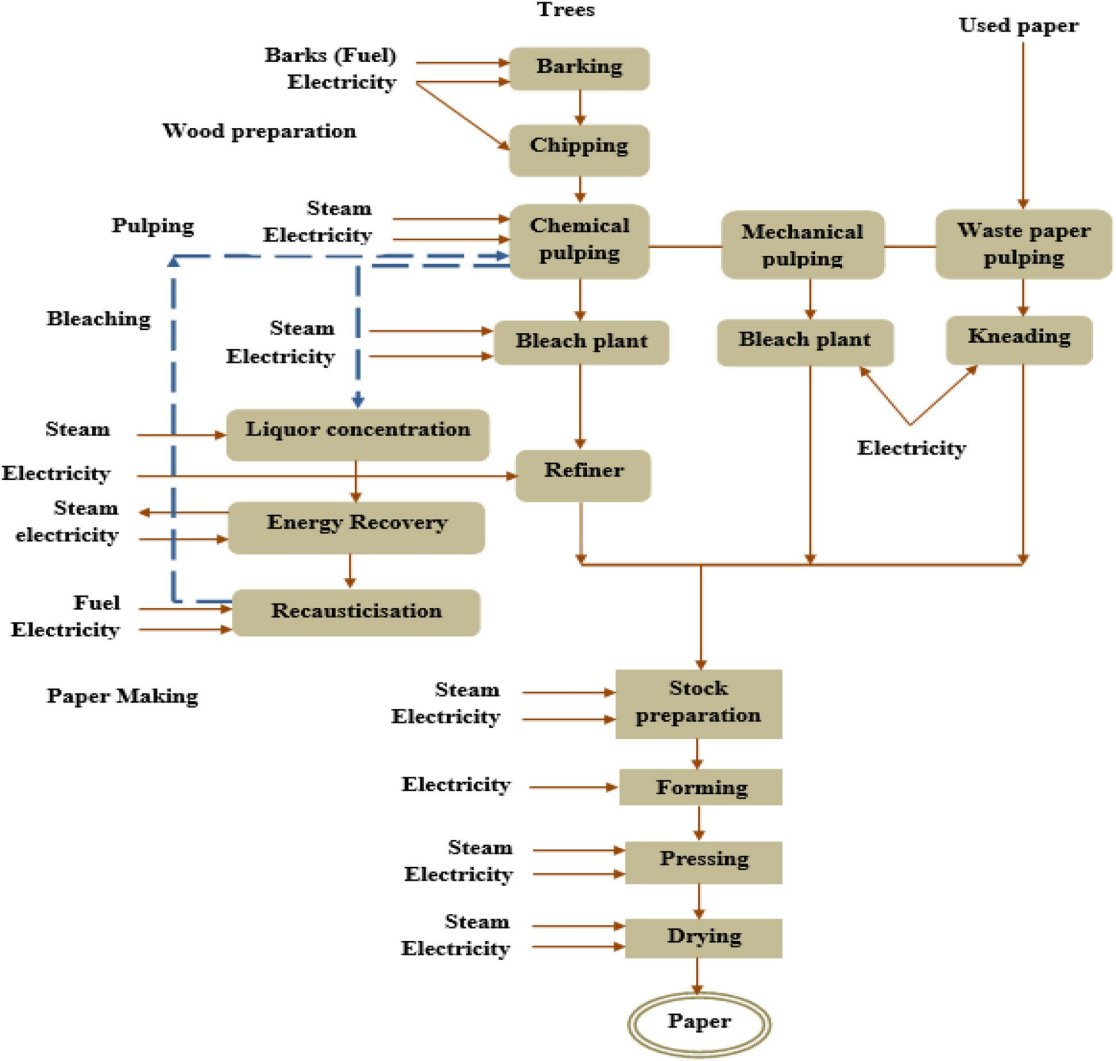
Process flow in pulp and paper manufacturing mills

The different pulping operations and the integrated paper manufacturing process are shown in Fig. 3. Two steps are involved in the manufacturing of paper. First cellulose sheets are converted into pulp which is the intermediate material for paper production. The second step involves the conversion of pulp into the paper with the necessary after-treatment depending on the targeted end-use. The overall manufacturing process is accompanied by multiple stages which mainly involve raw material preparation and handling, controlled pulping, and discrete or continuous paper manufacturing. The pulping involves the actual making of the pulp followed by pulp washing and screening. After proper chemical recovery and bleaching, the stock is prepared. Finally, the papermaking is carried out which itself has different stages. Pulp mills and paper mills may exist separately or as integrated operations (Bajpai 2018b; de Alda 2008; Ragauskas 2002).

### Operational mechanism of pulping

Pulp manufacturing starts with raw material preparation, which includes debarking, chipping, chip screening, chip handling, and storage (Bajpai 2011, Brännvall 2009). The pulp needs to be free of dirt and superficial materials such as bark, and debarking is done for making the pulp physically clean. The next operation in raw material preparation is chipping to ensure better handling of the logs which is done through cutting to get the required size of logs for the subsequent pulping process. Pulping requirements need to be considered in optimizing chip handling and determining the suitable chip storage conditions (Bajpai 2015a).

The pulping operation involves the separation of the wood chips to get the fibrous materials. In the process, lignin is removed from the lignocellulosic wood and the individual cellulose fibers are isolated. Though there are different pulping methods chemical and mechanical methods are the most widely used in paper mills. Either of the three techniques namely kraft, soda, or sulfite are used in the chemical pulping approach. In all the cases the cellulose is extracted via cooking of the chips in the aqueous solutions of the reagents at elevated pressure and temperature (Bajpai, 2011, 2015a). Physical energy is used in the mechanical pulping approach which mainly involves the removal of cellulose through grinding or shredding.

The most widely used pulping process is kraft pulping and uses alkaline cooking liquor of sodium hydroxide (NaOH) and sodium sulfide (Na_2_S) to digest wood. In the process, a digester is used for mixing the chips with the kraft cooking solution. The kraft pulping process uses an alkaline cooking liquor of sodium hydroxide and sodium sulfide to digest the wood. The process involves the digestion of wood chips at high temperature in the range of 145–170 °C and pressure in “white liquor,” which is a water solution of sodium sulfide (Na_2_S) and sodium hydroxide (NaOH), for a few hours (Huber et al. 2014). During this treatment, the hydroxide and hydrosulfide anions react with the lignin, causing the polymer to fragment into smaller water/alkali-soluble fragments and isolating the cellulose fibers.

After the wood chips have been cooked, the contents of the digester are discharged under pressure into a blow tank. As the mass of softened, cooked chips impacts the tangential entry of the blow tank, the chips disintegrate into fibers or “pulp.” The pulp and spent cooking liquor (black liquor) are subsequently separated in a series of brown stock washers (Alén 2019). After the required cooking is carried out the cooking liquor is discharged under pressure into a blow tank. The mechanistic tangential flow of the cooked chips in the blow tank isolates the chips into pulp (Yang and Liu 2005; Al-Dajani and Tschirner 2008).

After pulp production, after-treatment of pulp is carried out through washing to ensure removal of contaminants which include uncooked chips, and recycling of residual liquor is carried out via pulp washing operations. The pulp washers separate the pulp from the spent cooking liquor during chemical pulping (Dimmel and Gellerstedt 2009, Chakar and Ragauskas 2004). After washing, screening is done to remove remaining off-size particles such as fragments from barky matters, bigger size chips, and chips that remained uncooked, and the material is delivered to the pulp bleaching process.

A very important process in the pulp mill is bleaching used for the removal of coloring and allied impurities in the raw pulp. Bleached pulp grades are used to produce white papers. Nearly half of the Kraft production is in bleached grades. The bleaching of pulp is carried out to increase the whiteness and brightness of the pulp (Bajpai 2013; Hammett et al. 2001). This is important for making the paper suitable for selected products such as those produced in tissue and printing enterprises. Enhancement of physical and optical qualities of the pulp is achieved by removing impurities such as entangles of fibers, barky fragments, or decolorizing the lignin making it a potential benefit in the chemical pulping process (Al-Dajani and Tschirner 2008, Chakar and Ragauskas 2004).

Kraft bleaching has been refined into a stepwise progression of chemical reaction, evolving from a single-stage hypochlorite treatment to a multi-stage process, involving chlorine), chlorine dioxide, hydrogen peroxide, and ozone. The optimized and controlled condition must be used in the bleaching process. If bleaching conditions are too severe there will be fiber damage, leading to a lower strength of the paper (Brännvall 2009; Angevine, 1998). After bleaching stock preparation is conducted to convert raw stock into finished stock to be used in the paper machine. Raw stock can be available in different forms. In batch processes, the delivery can be in loose pulp or bale and the integrated pulp to paper conversion suspensions can be used as well (Dimmel and Gellerstedt 2009, 2009).

In the chemical pulping process, it is necessary to recover the used cooking chemicals, and a proper chemical recovery system is needed (Gellerstedt 2009; Walker 2004). At the pulping mills, the weak black liquor also called spent cooking liquor, from the different rinsing stages is transferred to the chemical recovery which as well is situated alongside the mills (Santos et al. 2011; Al-Dajani and Tschirner 2008). The chemical recovery process is conducted at different stages to ensure proper reconstitution of the cooking liquor. Initially, the weak black liquor is concentrated followed by combustion of organic matters and reduction of inorganic matters. Finally, the cooking liquor is reconstituted in the required concentration. All the stages need critical control on the parametric allowances. The quality of paper produced from the pulp is affected by the quality of water and different companies have incorporated water treatment schemes along with the pulping process. To prevent deposits on pulp, water treatment chemicals such as anti-scale agents and pitch control agents are incorporated during the route of manufacturing.

As part of pulping process dregs, lime mud, and grits are generated in the chemical recovery process. Green liquor contains sodium sulfide and sodium carbonate; and insoluble unburned carbon and inorganic impurities called dregs, which are removed in a series of clarification tanks. The green liquor is a residue of dissolution of the molten inorganic salts (smelt) from the black liquor concentration process (Angevine 1998, Brännvall 2009). The lime mud is generated in the causticization process of chemical recovery in the decantation of the green liquor using calcium oxide (lime) (Ai et al. 2007). The grits are originated from the white liquor slurry in the slacker by gravity and classified as unreacted lime (Kinnarinen et al. 2016). Grits are insoluble compounds, most of which come from purchased lime, which collect in the slacker and need to be removed and disposed of.

Final pulping operations are involved in the boiler unit which causes the generation of large content of ashes. In particular, fly ash is produced as by-products of the biomass combustion process which is generated during the high-temperature combustion of hog fuel in power boiler units (Cherian and Siddiqua 2019). As the flue gas approaches the low-temperature zones, the fused substances solidify to form fly ash. The fly ash consists of fine particulates and precipitated volatiles, typically with a high specific surface area, whereas bottom ashes tend to be coarser in texture (Cherian and Siddiqua 2019; Scheepers and du Toit 2016).

Most of the pulping operations so far especially those involving chemicals are not ecofriendly. Nowadays new pulping methods are used for manufacturing different grade pulps that utilize enzymes (Lin et al. 2018). These enzymatic pulping techniques are being used for the preparation of dissolving pulp for the production of fibrous materials. In the enzymatically assisted pulping process xylanase, cellulase, and hemicellulase enzymes are utilized for segregation of the pulp from the rest of lignocellulosic mass and after-treatment (Lin et al. 2018; Rashmi and Bhardwaj Nishi 2010; Yang et al. 2019b). It is reported that the purity of enzymatically produced pulp is better than conventional kraft pulping processes.

### Papermaking process

The process adopted for the making of paper is identical for all pulp types (Bajpai 2018b; Woiciechowski et al. 2020). The pulp from the chest compartment is screened which when required is refined to the required level. The pulp slurry is formed by mixing the refined pulp in water in a wet end operation. This slurry at the headbox is put through a paper machine and pressed by the press compartment. In the press section, the sheet forming process is commenced through the draining of the water. The next operation is passing of the formed sheet in a dryer which involved hierarchically arranged compartments of the dry end for coating and drying. The final dried and finished product is transferred to calendaring operation for reducing the thickness of paper as required and sheet surface smoothing before winding on to the take-up reels.

#### Availability of by-products from pulp and paper mill

A global review of manufacturing sectors divulged that 17% of the total global waste comes from paper industries (Fig. 5) (Karak et al. 2012; Sakai et al. 1996). Pulp and paper mills contribute to air, water, and land pollution and discarded paper and paperboard make up roughly 26% of solid municipal waste in landfill sites (Pati et al. 2008; Ritchlin 2012). Waste data based on relative waste analysis from a specific global company revealed the large contribution of pulp and paper mill waste to the total solid waste indicative of a need to set up a platform for strategic intervention for the realization of a green environment (Mladenov and Pelovski 2010; Ince et al. 2011). The study reveals that from a total solid waste generation of 238,771 thousand tonnes, around 186 thousand tonnes is generated from pulp and paper industries.

**Fig. 4:**
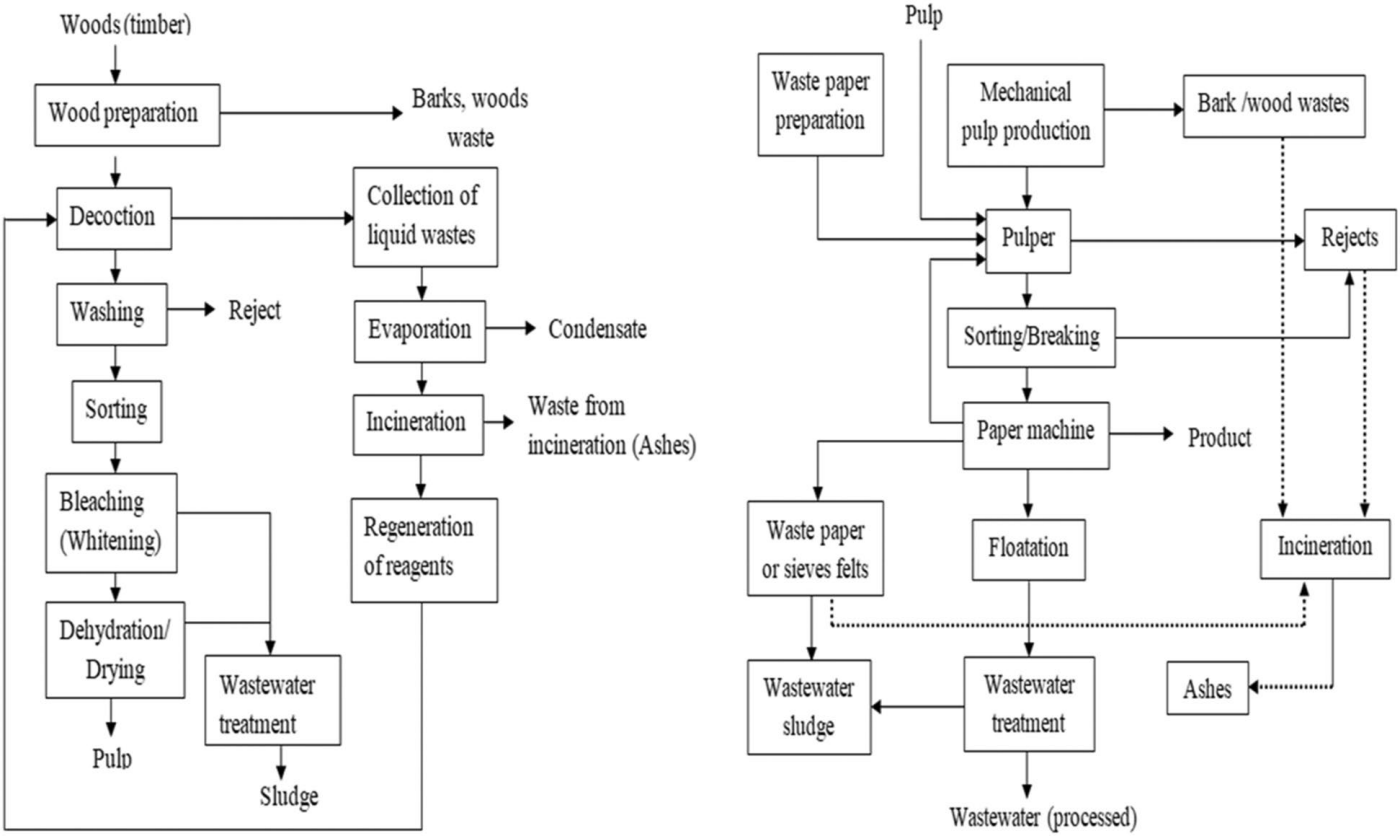
Sites of waste generation in pulp and paper manufacturing

Different types of solid wastes and sludge are generated in the pulp and paper mills at different sites of the process (Fig. 4). Potential wastes are generated from wood preparation and the actual pulp manufacturing and paper-making stages. Furthermore, an ample amount of different types of wastes are generated from chemical recovery, effluent treatment, and paper manufacturing methods that utilize recycled paper routes (Camberato et al. 2006; Akbari et al. 2018).

Among different factors that affect the identity and amount of generated waste from pulp and paper, mills is the type of manufacturing process amplified with the wastewater treatment technologies adopted. The pulp and paper mill waste mainly includes rejects at different stages such as woody and barky residues and sand particles, black liquor, and wastewater sludges. Inorganic sludges are isolated from the chemical recovery station and are composed mainly of calcite lime mud, the slacker grits, and green liquor dregs (Bird and Talberth 2008; Environmental 2007; Leponiemi 2008).

The sources of wastewater treatment residues as part of potential sludge are conveyed from two sources. The major part is the primary sludge from the entire manufacturing route and those generated from the secondary clarifier are categorized as biological sludge (Simão et al. 2019). Also, the sludge from the water treatment is regarded as chemical flocculation sludge. Deinking sludge from recycled paper production which contains mainly tiny fibers or fragmented fines and additives is another source of waste (Simão et al. 2018). The major source of waste from different stages of pulp and paper manufacturing is summarized in Table 3.

**Table 3:** Potential pulp and paper mill waste types

Type of waste	Source of waste
Black liquor	Chemical pulp manufacture (kraft process)
Wood/bark residues [Saw dust and others]	All types of pulping mainly chemical and mechanical methods of pulp manufacturing
Screening and other rejects	Stock preparation for paper machine, kraft pulping, and recycling route for paper manufacturing
Semi-mechanical sludge	Effluent and water treatment
Biological sludge	Effluent treatment utilizing biological technique
Sludge from de-inking treatment	Recycled paper de-inking treatment

The regions and different recycling processes and allied recycling rates determine the quantity of biomass generated from paper production. The variation within regions is also very wide. Due to internally established treatment and utilization routes for generated waste along with the manufacturing process by different pulp and paper mills, there is limited quantitative data on the total amount of waste generated from the mills. Estimated data on the quantity of solid waste from sample pulp and paper mill is reported. The potential woody wastes and rejects are from the preparation of pulpwood and contribute to solid waste generation (Table 4).

**Table 4:** Generation of solid waste from kraft mill (Gavrilescu 2004; Bajpai 2015b; Quina and Pinheiro 2020)

Type of waste	Yield (kg/t of dry pulp)
Sawdust	10–30
Bark	100–300
Pins and fine	50–100
Woody fragments/wood yard/	0–20
Knot	25–70
Recovery boiler salts	5–10
Dregs	10–30
Grits	15–40

The predominant biomass available in large quantities in pulp mills is the black liquor. It is reported that kraft mill producing bleached paper engenders around 1.7–1.8 tonnes of black liquor per one tonne of pulp on a bone dry basis (Leponiemi 2008). This highly viscous liquor is a result of the conversion of almost half of the raw chips used in the hopper of the pulping operation. A report of IEA revealed about 170 million tonnes of black liquor per year is released from pulp and paper mills worldwide.

Another portion of the waste involves sludge of different types namely primary, secondary, and deinking sludge. From a total sludge of 40–50 kg per tonne of the virgin paper production, the primary sludge accounts for 70% and the rest 30% is the secondary sludge. On a glance approximation, the sludge generated from the manufacturing of different paper products varies from 20 to 40% on a dry mass. The grade of paper and the specific type of process adopted for the manufacturing of the paper product determines the amount of sludge generated. (Quina and Pinheiro 2020). In comparison, the amount of waste sludge generated from industries that utilize the recycled paper approach produces a large amount of sludge than the conventional approach through virgin raw materials. From each one tonne of reclaimed paper, a total of 300 kg sludge is generated.

Depending on production capacity and the type of pulping a large amount of fly ash is released annually from pulp and paper mills (Environmental 2007; Mikkanen 2000). A recent report in Finland indicated a release of 240,000 tonnes of ash every year. As an illustration of the availability of waste biomass from pulp and paper mills, it is reported that the collective fly ash and wood ash produced from Canadian mills is 1 million tonnes per year.

Most of the pulp and paper mill wastes have an impact on the environment and create health problems. The major sources of pollutants at different stages of pulping and paper manufacturing are summarized (Table 5) (Gavrilescu et al. 2012). The environmental impact generated by the manufacture of pulp and paper results mainly from wood pulping and pulp bleaching. In pulping processes, sulfur compounds and nitrogen oxides are emitted into the air, and during pulp bleaching, chlorinated and organic compounds and nutrients are discharged to the wastewaters (Gavrilescu et al. 2012; Singh and Chandra 2019).

**Table 5:** Potential pollutants from pulp and paper mill waste

S. N	Main input	Process step	Pollutants
1	Raw material/wood	Wood preparation	Solid wastes and wastewater
2	Chemicals/energy	Pulp manufacture	Air emissions and used water
3	Process water	Pulp washing/screening	Dissolved material, residual chemicals, and wastewater
4	Chemicals/energy	Pulp bleaching	Air emissions, dissolved material, residual chemicals, and wastewater
5	Energy	Pulp drying	Air emissions
6	Energy/water/chemicals	Paper manufacture	Solid wastes, dissolved material, residual chemicals, and wastewater

The pulp paper industries release very complex organic and inorganic pollutants mainly from pulping and bleaching stages. Particularly, sulfur compounds and nitrogen oxides are emitted into the air, and chlorinated and organic compounds and nutrients are discharged to the wastewaters (Gavrilescu et al. 2012; Bank 1999). The pollutants are gaseous, inorganic, and organic type. The major pollutants include hydrogen sulfides, sodium sulfide, sulfur, chlorine dioxide, ferrous, copper, hexadecanoic acids, octacosane, phenol, and β-sitosterol. It has been reported that the different pollutants cause environmental and health impacts in terms of respiratory disorder and irritation to the skin, neurotoxicity, and direct toxicity of effluent to the reproductive system in aquatic flora and fauna are reported. Several of these pollutants are reported to contain endocrine-disrupting chemicals. Furthermore, the environmental pollution caused by the pollutants poses a threat to aquatic life as well as to plants and human beings (Bank 1999; Gavrilescu et al. 2012; Singh and Chandra 2019)**.**

## Current utilization of pulp and paper mill waste

### Characteristic components of major by-products

**Fig. 5:**
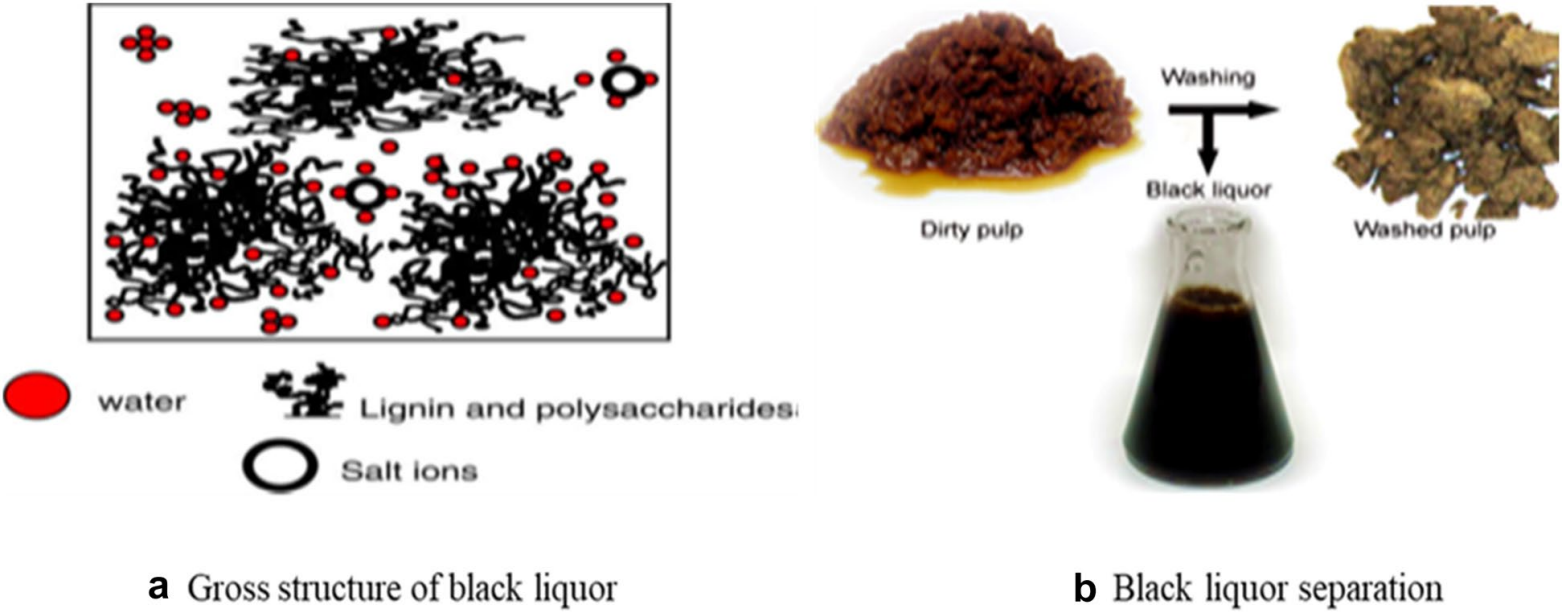
Black liquor: **a** Gross structure of black liquor; **b** Black liquor separation

The wastes from pulp and paper mills are both of organic and inorganic types. Most of the biomass generated from pulp and paper mills is organic residues. Black liquor consists of organic polymers (lignin, polysaccharides, and resinous compounds of a low molar mass) and inorganic compounds mainly soluble salt ions (Fig. 5a) (Viel et al. 2020).

Black liquor is separated from the pulp in washing (Fig. 5b). The biorefinery approach can be used for the conversion of lignin and hemicellulose in black liquor into biomaterials (Vakkilainen and Välimäki 2009; Vakkilainen 2016).

**Fig. 6:**
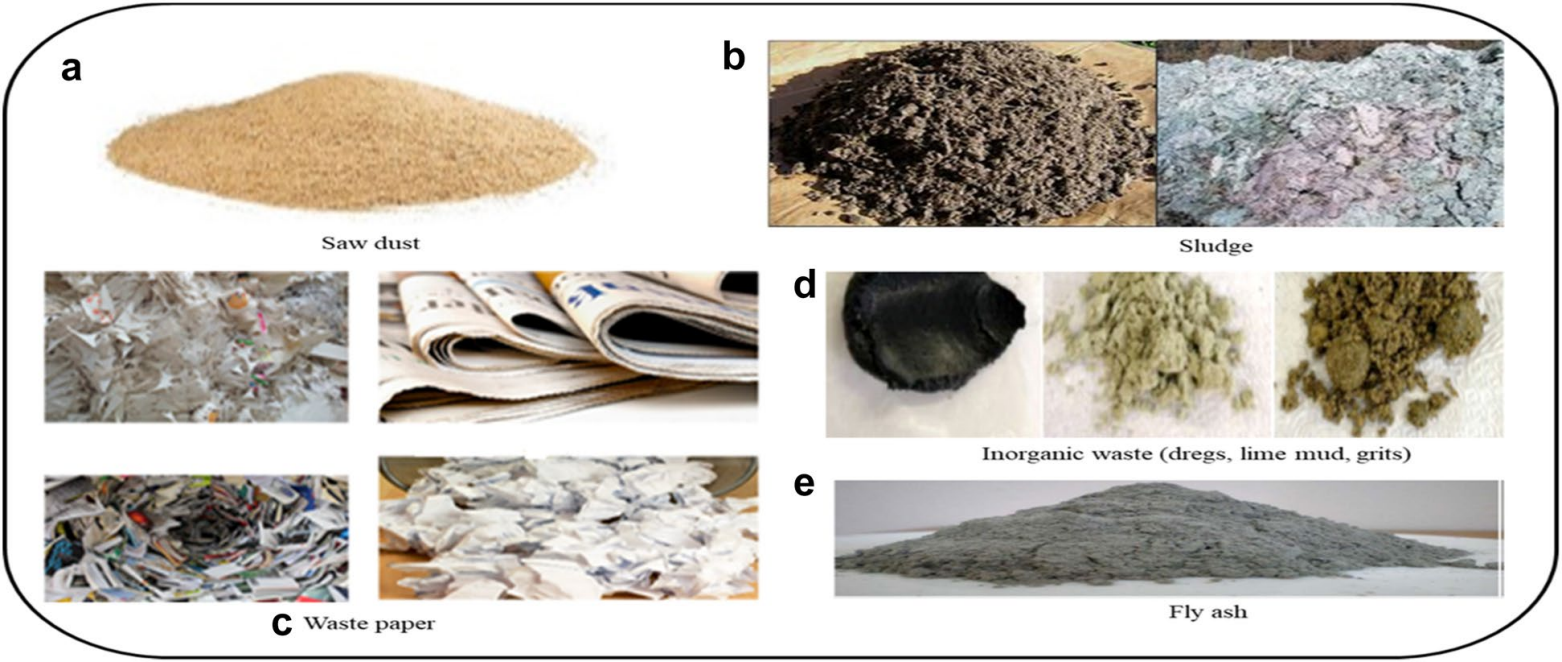
Major wastes generated from pulp and paper mills: **a** sawdust; **b** sludge; **c** waste paper; **d** inorganic wastes (dregs, lime mud, grits); **e** Fly ash

Black liquor being the major waste other potential wastes include sawdust, sludges of different types, waste paper, and fly ash. The major inorganic type wastes from pulping and after-treatment stage include the dregs, lime mud, and grits. The different wastes are obtained as extracted from different pulp and paper industries are summarized (Fig. 6).

The sawdust (Fig. 6a) as woody material is composed of cellulose, lignin, and hemicelluloses (Bajpai 2018a). The cellulose composition of sawdust is by far higher than the lignin content. Accordingly, the interest in biomaterial synthesis relies on the cellulose component of the sawdust. The current preferably adapted extraction of cellulose from sawdust involves the preparation of sawdust (drying, cutting, and conditioning); dissolution of wood in appropriate ionic liquid, and precipitation of cellulose through the removal of lignin.

Another potential source of cellulose is wastewater treatment plants and recycled paper converters. The virgin or waste recycling sludge (Fig. 6b) constitutes cellulosic materials. The mill sludge accounts for the main waste from pulp and recycled paper production. Pulp and paper mill sludge is an organic residual generated from wastewater treatments (Faubert et al. 2016). Per unit of paper production 23.4% sludge is generated. The major components of the paper mill sludge are inorganic residues and organic cellulosic fines and fragments (Albuquerque et al. 2015; Abdullah et al. 2015). The organic constitutes in primary sludge mainly consist of cellulosic fibers.

A noticeable quantity of cellulose can also be obtained from waste paper (Fig. 6c) which is a release of every paper utilizer with a huge amount from office and related service providers. Directly or as part of recycling residue, the waste paper is considered as a major source of cellulose, and using appropriate cleaning and recycling procedures the cellulose can be made use of as a raw material for biomaterial synthesis (Kim and Kim 2019).

Inorganic wastes (Fig. 6d) namely liquor dregs, calcite mud, and slacker grits are also considered as the major portion of solid waste in the mills (Quina and Pinheiro 2020). These mineral wastes are generated during the chemical recovery process at the pulping mill. The green liquor dregs are obtained The inorganic biomasses contain different types of minerals (Bird and Talberth 2008; Leponiemi 2008). The mineral Gipsite and Calcite are present in the dregs and the mud, whereas the minerals such as brucite, larnite, pirssonite, portlandite, and calcite are ingredients of the biomass grits. The mineral content of these wastes can be made administered and used for making biomaterials.

The successive development in the pulp and paper mill has headed to the release of a large quantity of fly ash (Fig. 6e) as a result of combustion processes of waste biomass. The elemental composition survey on fly ash from pulp and paper mills revealed that the ash consists of different minerals predominantly iron oxide, silica, and alumina. The fly ash also consists of other metal oxides such as oxides of sodium, calcium, magnesium, potassium sulfur, and titanium (Na_2_O, CaO, MgO, K_2_O, SO_3,_ and TiO_2_) in variable quantities. Through polymerization, the metal oxides can be converted to biopolymers for diversified end-use. The widely and adopted and conventionally established way of valorization of fly ash is geo-polymerization. In the polymerization process demand-driven three-dimensional aluminosilicate and similar materials so-called geopolymers are synthesized (Cherian and Siddiqua 2019; Sjöström and Westermark 1999).

### Disposal technique

#### Waste management in pulp and paper mill

The pulp and paper mill generates a large volume of wastes with an estimate of around 100 tonnes per 550 tonnes of pulp production. Though the toxicity of pulp and paper wastes is minimal appropriate disposal technique is required for appropriate management of the land, environment, and allied issues (Monte et al. 2009). As per the environmental policies recommend based on directives of the waste framework the landfill must be reduced by avoiding disposal of waste from industries. In certain cases, an obligation is placed for selected wastes to terminate as a waste resource. The wastes from pulp and paper mills have a tremendous negative and adverse impact on the environment. Inherently high consumption of water is the major cause, and the problems are wider in terms of generation of effluent and other wastes of solid and liquid type. These solid and liquid wastes along with air emissions require effective disposal and treatment approach (Bajpai 2015d).

Three different approaches can be used in pulp and paper mill waste management. The first choice is to minimize waste in ensuring product efficiency, higher yield of materials, and lower waste management (Mladenov and Pelovski 2010). The second choice is to find a suitable way for reuse to maintain cost improvement and lower environmental approach. This could be done by material or energy valorization of the generated wastes. The final option is landfilling which shall only be done when a choice is not an option.

In minimizing waste generation, it is necessary to cope up with established concrete environmental legislations. In line with this concerns related to cost of disposal, treatment technologies, and opt for new utilization schemes need to be taken into consideration. Different techniques are adopted for the reuse of the mill wastes which include burning or incineration, biofuel production, gasification, pyrolysis, and anaerobic digestion (Bajpai 2015d; Mladenov and Pelovski 2010). As far as the application is concerned; for solid wastes incineration techniques is widely used, whereas anaerobic digestion is widely practiced for wastewater. Landfilling is still the most widely adopted disposal technique.

#### Current trends in the disposal of pulp and paper mill wastes

In the current world pulp and paper mill practice, both landfill and incineration techniques are applied for the disposal of waste. The opt for incineration and other reuse options differ from country to country and are based on technology availability and economic growth hierarchy (Ince et al. 2011; Bahar et al. 2011). Most of the solid wastes generated from pulp and paper mills are disposed of as landfills. Landfilling with its critical limitations regarding increasing volumes and the possibility of hazardous substances is still the most widely used disposal technique by pulp and paper mills. The hazardous matters in the watercourse impose environmental dangers as well. As far as the context in most of the developing countries is concerned most of the waste is still landfilled and some are incinerated.

The black liquor is incinerated or gassed as a source of fuel energy and where the technology is not available it as well is discarded as a landfill. Rejects are dewatered and burnt for energy recovery (Bahar et al. 2011; Naqvi et al. 2010a). Other wastes like sawdust and woody matters are also discarded in the landfill. The destination of liquor dregs, grits, lime mud, and mill ash is the landfill after dewatering and drying.

The wastewater sludge is disposed of through a burning approach. The conventional sludge treatment process includes the thickening of the sludge waste which is dehydrated by mechanical means. The prepared sludge is incinerated at optimal conditions. In the pretreatment process blending of sludges is done with the addition of polymer and dewatering is carried out to obtain a dry solid content of 25–40%. Incineration which is the widely practiced internal route for biofuel production in pulp and paper mills is carried out. The deinking sludge from recycled paper production can be incinerated or reused in other mills (Faubert et al. 2016).

### Current uses of pulp and paper mill waste

The pulp and paper mill waste have different chemical constituents and physicochemical characteristics. Based on their chemical and physical properties the wastes are widely used in conventional and industrial applications. As most of these wastes are mainly based on woody features comprising lignocellulosic behavior they are used as fuel or energy sources for pulp and paper mill or other industries (Simão et al. 2018; Sarkar et al. 2017). Many are used for construction and building as potential and economic substitutes. The major schemes and sectorial applications followed in the current utilization of wastes from pulp and paper mill are summarized (Table 6).

**Table 6:** Current uses of pulp and paper major wastes

S. N.	Type of waste	Utilization scheme	Industry
1	Black liquor	Energy	Pulp and paper
2	Sawdust	Furniture and building	Construction
3	Sludge	Compost	Agriculture
		Energy	Pulp and paper
		Cement base	Construction
4	Fly ash	Binder Components	Construction
		Soil Amendments	Agriculture/Forestry
		Cementitious Material	Construction
5	Dregs	Fertilizer	Agriculture
		Wastewater treatment	Environmental technology
6	Grits	Building	Construction
7	Lime mud	Fertilizer	Agriculture
		Building	Construction
		Stabilization	Environmental technology

The incinerated black liquor in recovery boilers is used in pulp and paper mills as an optional energy source as it provides steam. It is also possible to reclaim pulping chemicals for reuse. The current and alternative technology for reuse is the gasification of black liquor. This process has the potency to replicate the amount of production energy used in the pulp and paper mill (Naqvi et al. 2010b). The sawdust as well can be used as a secondary raw material for pulp production and recycled for paper manufacturing (Srinivasakannan and Bakar 2004). It can also be used as filler for the manufacturing of bricks and cement material in the field of construction.

The sludge from pulp and paper mills can be recycled via different routes. Mainly the sludge can be used in construction and energy and as a chief source of energy. The suitability of sludge for utilization in power generation is the inherent compounds present in the sludge that are organic with a high degree of combustibility.

Commonly cement plants use fly ash as their raw material together with other ingredients. Fly ash is also used as fertilizer in modern agricultural practice. The suitability especially in cement application is due to the inherent high strength to weight ratio of fly ash as compared with other cement materials. This is also supplemented by carbon footprint and minimal energy consumption which makes fly ash a replacement for cement in cost-effective construction and eco-efficient geotechnical applications (Cherian and Siddiqua 2019). It can also be used as an immobilizer of pollutants. Their physical properties such as mechanical resistance and durability make the fly ashes used in concrete systems utilized in hostile environments.

**Fig. 7:**
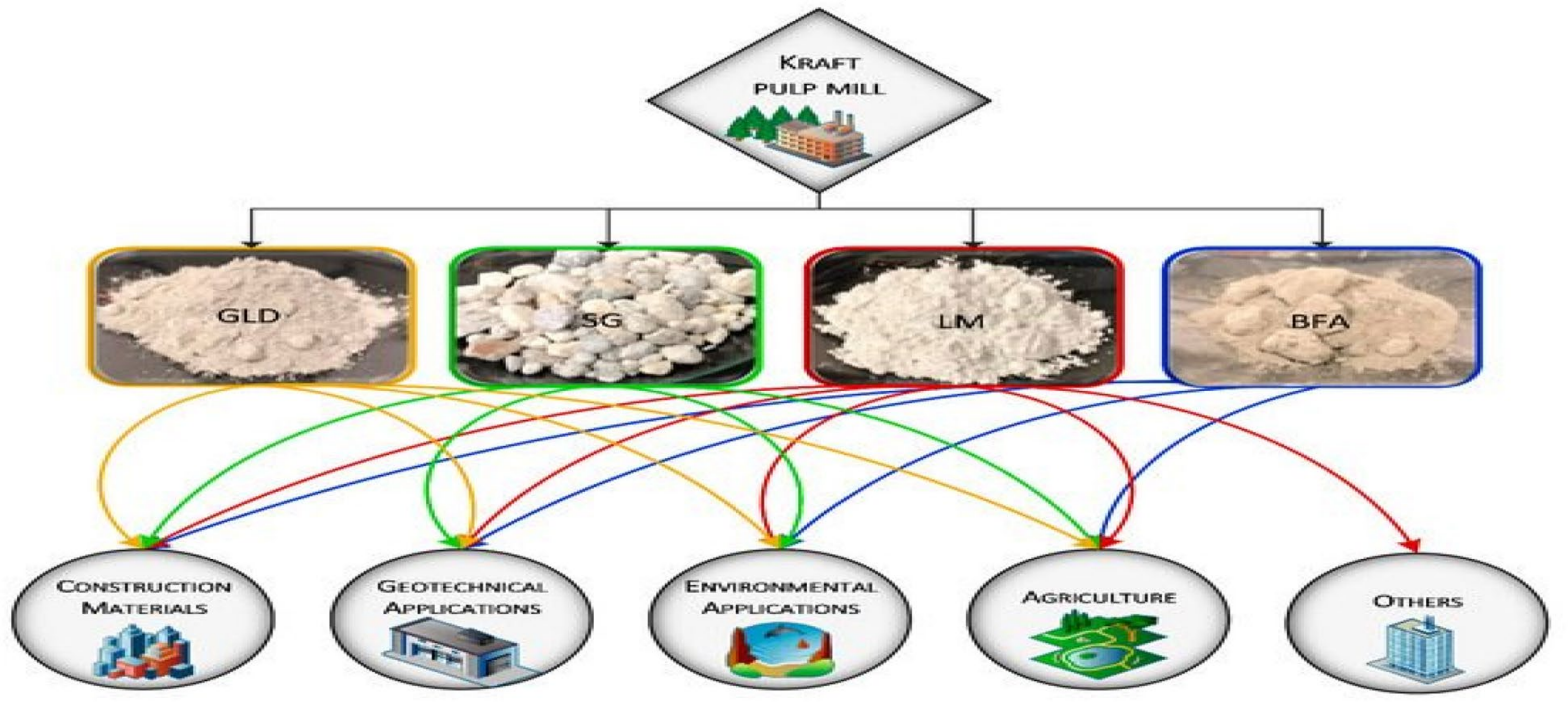
Current utilization of pulp and paper mill inorganic wastes (*GLD* green liquor dregs, *SG* slacker grits, *LM* lime mud, *BFA* boiler fly ash)

The inorganic wastes from pulp and paper mills have been engineered for different applications (Fig. 7). Green liquor dregs have shown promising effects for correcting soil acidity and fertilizer. Dregs have also been used in wastewater treatment. The inorganic grits are mainly composed of calcium carbonate and can be used for the replacement of calcareous raw materials such as in building and construction (Quina and Pinheiro 2020).

Agriculturalists already investigated the lime mud can be used in soil healing such as for fertilizing and remedial agents. Compositionally lime mud is similar to the commercially available calcium carbonate and can be used as a potential substitute and replacement in building materials. The crude carbonate component of lime mud provides high alkalinity which makes the use of lime mud in precipitation and immobilization of heavy metals in watery streams, removal of phosphorus, and stabilization of sewage sludge.

### Utilization of pulp and paper waste: future prospects

Most of the pulp and paper mill wastes in developing countries adopt recycling for lower grade paper; recycling being the only used pulp and paper mill waste utilization method. The scope and technology for utilization of the different wastes for other industrial applications are practically not available and not attempted (Raut et al. 2012; de Alda 2008). The biomass from pulp and paper mill is composed of different ingredients. The major wastes of biomaterial concern from these mills are black liquor, woody residues including sawdust, sludges of different types, and fly ash.

An abundant quantity of the mill wastes is disposed to landfill, and some are incinerated. Instead using an appropriate biorefinery approach, it is possible to extract different ingredients from the biomass for versatile utilization as a biomaterial in different fields of applications. Through the biorefinery approach environmental pollution is reduced or eliminated, product substitution is enhanced. Furthermore, the allied spinoff company for biomaterials and the industrial-scale prospect will boost the economic growth. The potential ingredients of current concern from the biomass are identified (Table 7). Based on the characteristics of ingredients in each biomass potential beneficiation routes are proposed for high value-added materials (Simão et al. 2018; Rajput et al. 2012).

**Table 7:** Potential ingredients available from pulp and paper mill waste

S. N	Type of waste	Potential ingredients
1	Black liquor	Lignin, xylan
2	Sawdust /woody residues	Cellulose
3	Sludges	Cellulose
4	Fly ash	Minerals

The general challenges with regards to the forecasted application of pulp and paper industry wastes as high value-added materials can be broadly classified into three patterns. One major challenge is the lower yield from each biomass in terms of the continuous supply of certain waste types. The lower yield of biomaterial ingredients from selected biomass can be enhanced by the integration of pulp and paper industries especially in developing countries like Ethiopia. Another challenge which is still prominent in developing countries is the availability of suitable and integrated waste to energy and related biorefinery system (Ismail and Nizami, 2016; Diep et al. 2012). This directly affects the mass production of high valued-added materials from pulp and paper industry biomass. The other potential challenge is the adequacy of labor in terms of skill as the production of the high value-added biomaterials requires stringent control allied with health and environmental safety concerns and regulations. There are also specific challenges in terms of each high-value-added biomaterial addressed in the review. These challenges need critical investigation and can be amended with versatile possibilities as far as continuous marketability of the biomaterials and their high value-added application is targeted.

### Beneficiation of black liquor for high value-added materials

#### High-performance carbon fiber from black liquor lignin

**Fig. 8:**
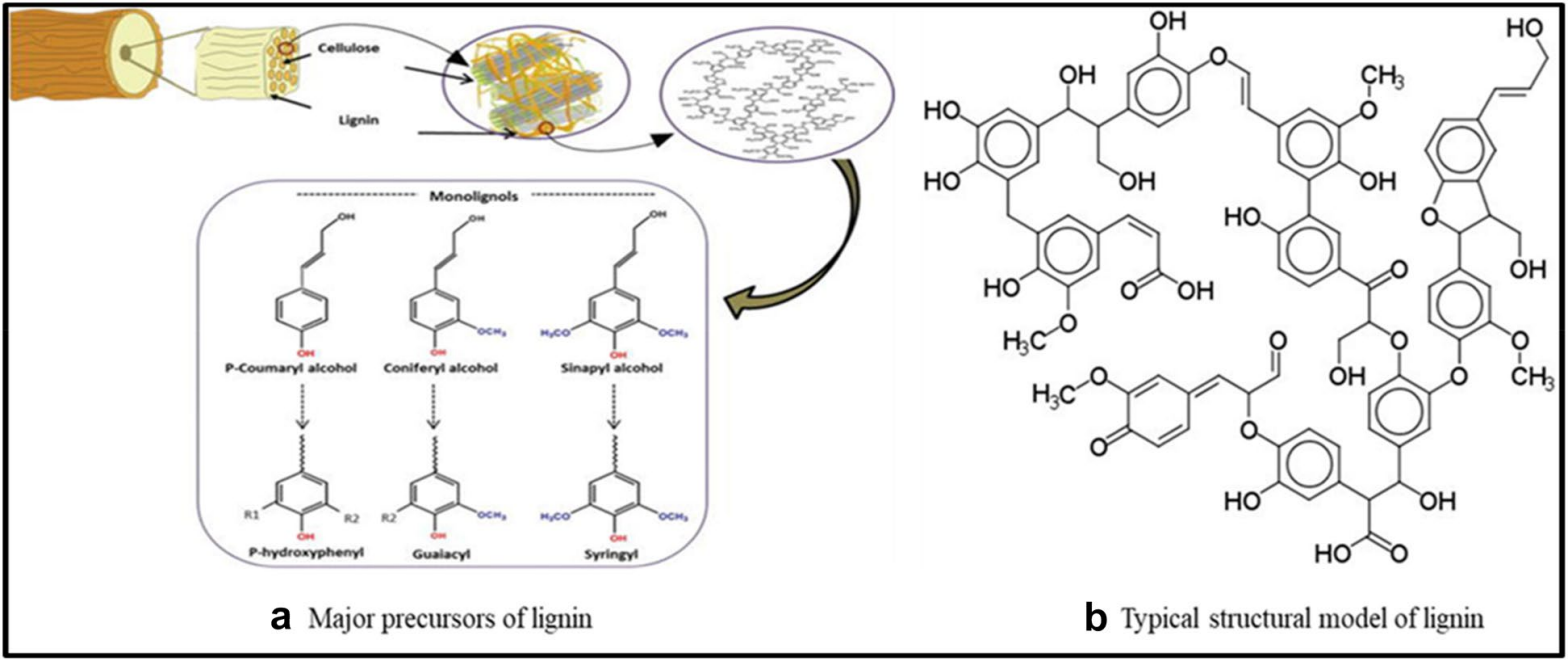
Major precursors of lignin (**a**) typical structural model of lignin (**b**)

There is an estimated 70 million tonnes of lignin from pulping processes worldwide (Dessbesell et al. 2020; Bajwa et al. 2019). Structurally lignin is a heterogeneous aromatic polymer and consists of mainly three precursors: p-coumaryl alcohol, coniferyl alcohol, and sinapyl alcohol (Fig. 8) (Chen 2012). It is a natural macromolecular with multiple aromatic ring compounds consisting of hydrophobic non-polar substructures of phenyl propane and polar groups like carboxyl.

Several approaches have been used to purify the spent black liquor for the production of pure lignin (Hubbe et al. 2019; Xinde 1996). Acidification via precipitation is the most prominent. The extraction involved precipitation of the black liquor in acid solution, coagulation, and removal of lignin (Norberg 2012; Olsson et al. 2017; Bengtsson et al. 2018).

The biomaterial options for lignin are versatile. The modern interest in carbon fibers is very crucial, and lignin is one of the important biomasses for the production of carbon fibers. Carbon fiber is known for its extremely high strength-to-weight ratio which makes it an ideal engineering material for a load-bearing element in lightweight high-performance composites. The versatile application of carbon fiber in the modern industry especially in composites is related to its low density and excellent mechanical properties.

High-performance carbon fibers are currently produced primarily from polyacrylonitrile (PAN). Carbon-rich precursor fiber with a carbon content of more than 90% is required for the manufacturing of carbon fibers. Once the precursor is availed the transformation is carried out in a two-stage thermal process. The delicate and rigorous web spinning process used for polyacrylonitrile-based precursors is one of the bottlenecks in the cost-effective manufacturing of carbon fiber. Half the cost of manufacturing carbon fiber is spent in making the PAN precursor (Bengtsson et al. 2019). Another potential limitation of using PAN precursors is the raw materials are fossil-based which entails a high cost. The high price of carbon from the perspective of raw materials and production cost is the driving force of finding cheaper and renewable alternatives.

**Fig. 9:**
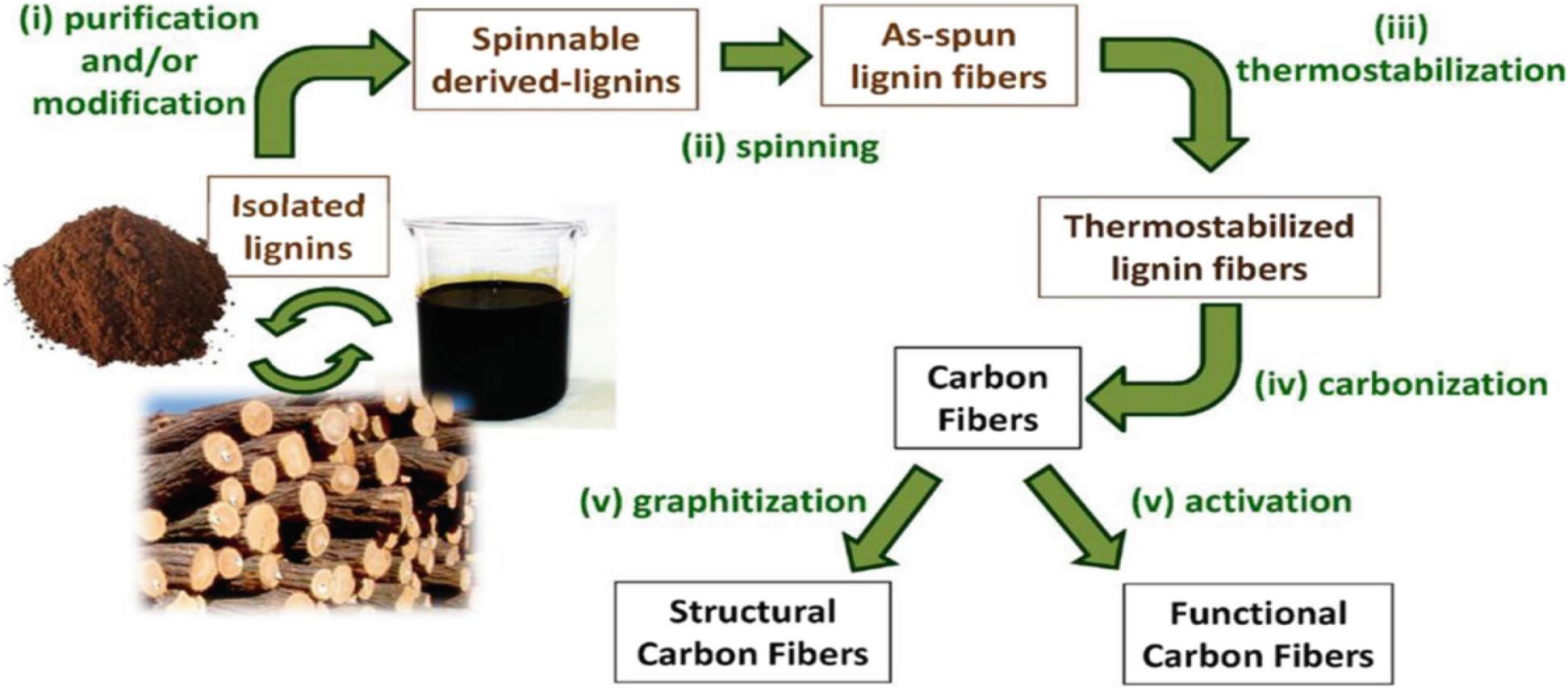
Spinning of lignin into carbon fiber (Gbenebor and Adeosun 2019)

A potential and interesting substitute precursor for carbon fiber production is lignin. It can be utilized for the production of a high volume of carbon fibers for a multitude of applications. This is possible, because especially the lignin from kraft pulp consists of high carbon content of around 60–65% and the lignin is available in huge quantity. This also makes lignin a promising alternative precursor based on high yield after processing into carbon fiber (Olsson et al. 2017; Hubbe et al. 2019). The lignin can be used as a potential precursor for carbon fiber production by incorporating appropriate spinning and carbonization processes. The most common way of manufacturing carbon fibers from lignin involves melt spinning to produce precursor fibers, thermo-stabilization (200–350 °C), and carbonization (over 1000 °C under nitrogen atmosphere) to produce carbon fibers (Fig. 9). Wet spinning can also be used when rheological problems are encountered in melt spinning. It is reported that greater than 35% of manufacturing cost is reduced using renewable lignin as a precursor in carbon fiber production (Bengtsson et al. 2018; Gbenebor and Adeosun 2019). The challenges in regards to lignin to carbon fiber conversion are the availability of fiber spinning technologies (Souto et al. 2018) in some countries which limit the product diversification scheme.

#### Bioplastics from black liquor hemicellulose

The hemicellulose from black liquor consists of xylan among other ingredients (Yang et al. 2019a; Liu et al. 2012). The polysaccharide-based xylans are made up of β-1,4-linked xylose residues. They consist of α-arabinofuranose and α-glucuronic acids pendant branches that contribute to cross-linking of cellulose microfibrils and lignin through ferulic acid residues.

Xylan can be extracted from several locations in the pulp mill. During kraft pulping, xylan is partly dissolved in the cooking liquor but part of it will be redeposited onto the fibers in the later parts of the cook. The dissolved xylan can be isolated from black liquor for its utilization in the production of different biomaterials. Among the different methods used for xylan extraction, those utilizing alkali are most commonly and widely used.

Xylan is constituted by the black liquor along with fractions lignin fractions. The extraction of xylan from black liquor requires two-step precipitation. First, black liquor acidification is conducted and this is followed by precipitation by ethanol which ultimately provides the fractions of xylan (Stoklosa 2014). The xylan hemicellulose separation is done using potassium hydroxide (KOH) for hardwood and sodium hydroxide for softwood in aqueous media. The mechanism of separation of the hemicellulose is achieved through hydrolysis which involves the breakage of ester linkages between xylan and other alkaline black liquor components.

**Fig. 10:**
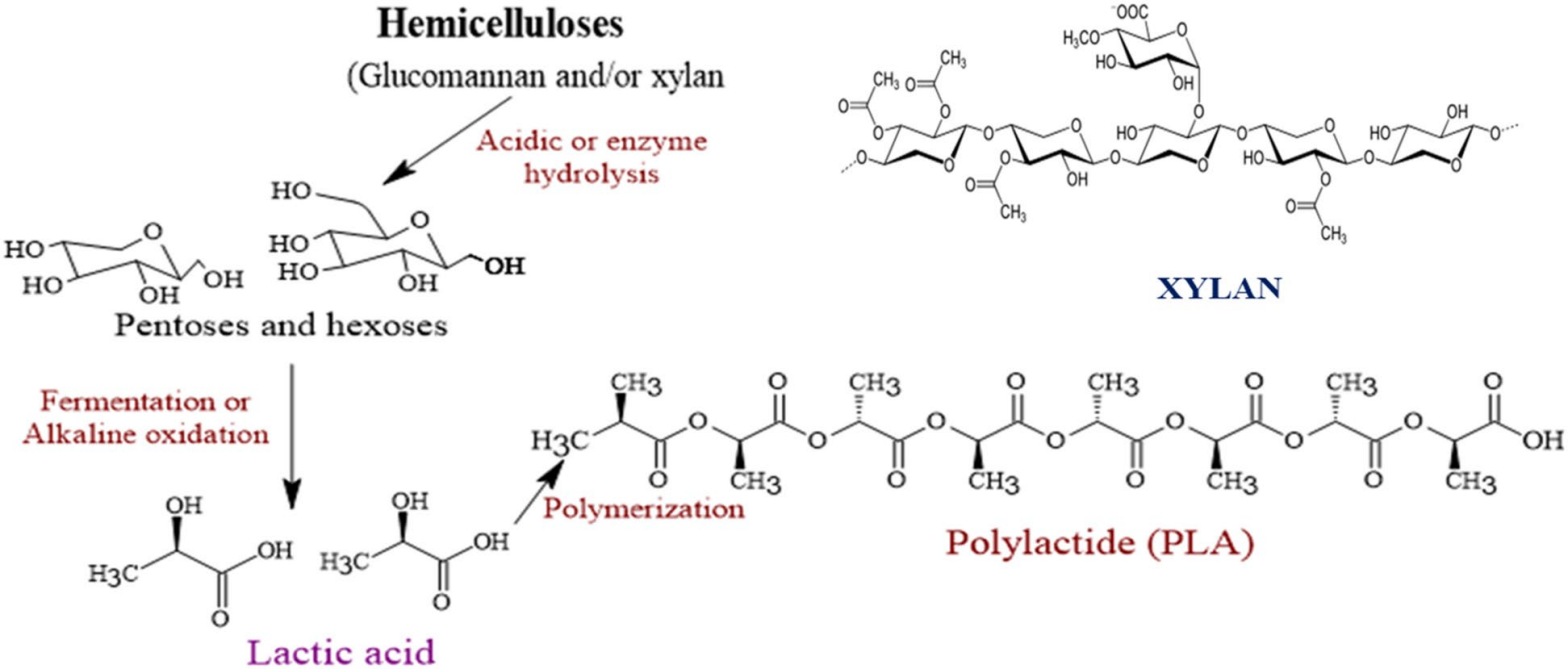
Xylan to PLA route

Xylan can be biorefined into lactic acid (Fig. 10). First, xylan is converted to low molecular weight sugars by hydrolysis using enzymes or chemically using acid hydrolysis. Then the resulting low molecular weight sugars can be converted to lactic acid by fermentation or alkali oxidation. Biomass-derived lactic acid is an important renewable chemical building block for synthesizing bioplastics (Fernández-Rodríguez et al. 2019; Chen 2012). Polylactic acid (polylactide) used as a precursor for plastics can be obtained by polymerization of a lactic acid refinery of xylan.

Polylactic acid (polylactide) [PLA] is biodegradable as well as recyclable polyester made from renewable feedstock. PLA is synthesized by the condensation polymerization of lactic acid or ring-opening polymerization of the corresponding lactide. As one of the highly biodegradable and crystalline polymers the lactic acid polymer (PLA) has a high melting point and outstanding mechanical characteristics (Gordobil et al. 2015; Singhvi et al. 2019). It can be used for bioplastic manufacturing used for different engineering applications. Biomaterials of versatile need can be obtained from PLA using advanced manufacturing techniques such as electrospinning, especially for medical products. The major challenges in conversion and utilization of xylan are of two ways. One is the difficulty in obtaining fully purified xylan with the grade required for polymer production and the other is the lower melting pint of the PLA produced which limits its application for high-performance materials. Different researches are ongoing especially in maximizing extraction effectiveness of xylan and usage of modified polymerization and spinning technologies for manufacturing higher grade PLA products.

### Beneficiation of cellulose for high value-added materials

#### Production of cellulose nanocrystals [CNC] from cellulosic residues

**Fig. 11:**
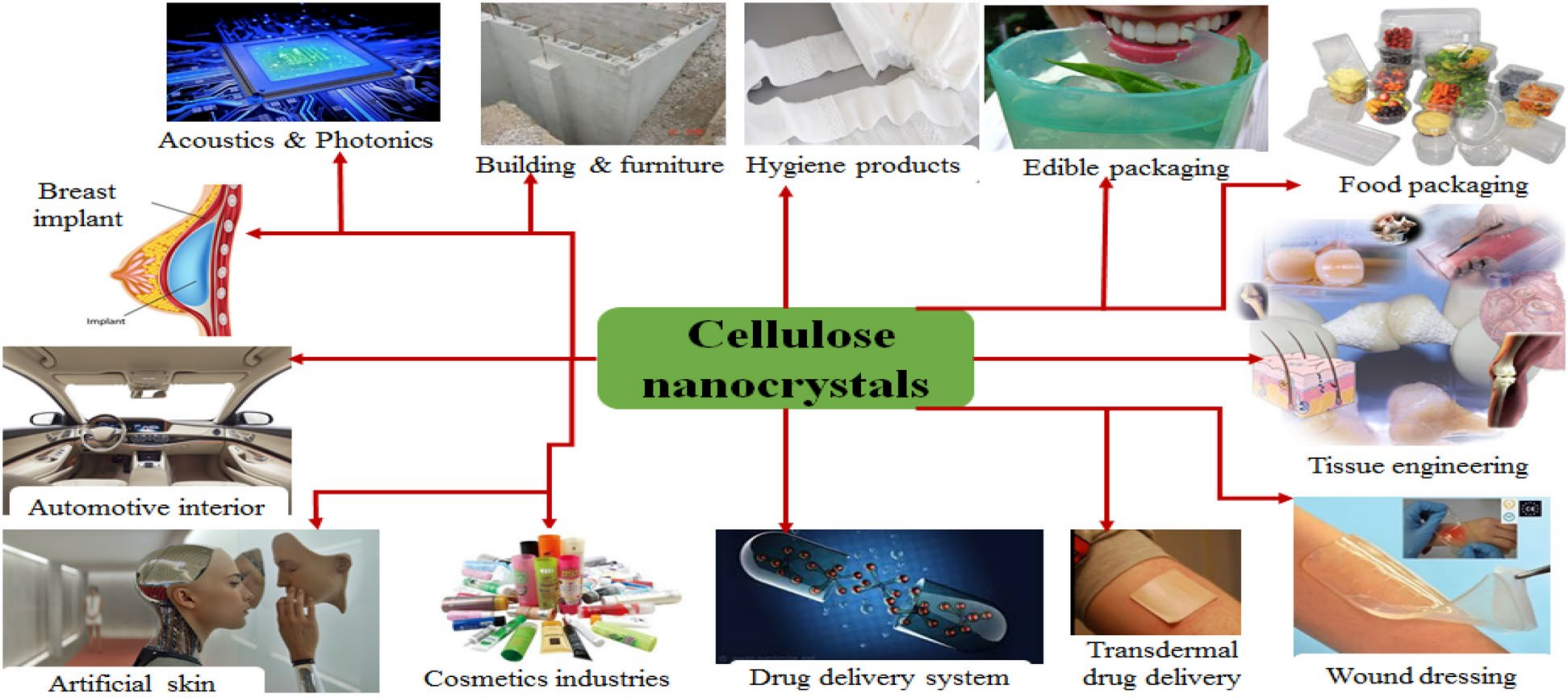
Multitude application of CNC biomaterial

Nanocellulose is a unique and promising natural compound derived from ordinary biomass. It is currently the most environmentally friendly compound that is techno-feasible and cost-effective, and also reduces effluent production (Clemons 2016). Nanocellulose has retained significant attention due to its tremendous functionality, i.e., greater surface chemistry, extraordinary biotic possessions, low toxicity, low cost, lower density, and significant mechanical properties. Cellulose Nanocrystals have many important physical properties Nanocrystalline cellulose has high heat stability which makes it suitable as a potential engineering material for aggressive temperature environments and its morphology with regards to small size and shape can be managed for different applications in solutions (Feng et al. 2015; Aguayo et al. 2018). The cellulose from the different waste sources in pulp and paper mills can be converted into cellulose nanocrystals (CNC) for wider application as a biomaterial in diversified fields of application (Fig. 11).

CNC is needle-shaped and highly crystalline material produced from cellulose pulp (Clemons 2016). The outlooked application of CNC is very wide and mostly its use in medical and industrial fields is tremendous. CNC can be used for the fabrication of medical products such as artificial skin, breast implants, versatile hygiene products, tissue-engineered materials, wound dressings, and drug delivery biomaterials. Industrially CNC can be used for the automotive interior, cosmetics industry, acoustics/photonics, build-tech, and different packaging materials.

Cellulose fiber is a key and characteristic component in pulp and paper mill sludge and is also constituted by sawdust and other woody residues (Aguayo et al. 2018). Cellulose is an important biopolymer that consists of semicrystalline regions which are responsible for the outstanding mechanical properties of the polymer. Cellulose as macromolecule is composed of many cellobiose repeat units which themselves are made up of glucose monomeric units (β-glucose) via β-1,4-glycosidic linkages. The individual extended cellulose chains are parallel to each other and the inherent stability and crystallization of cellulose polymers are due to the presence of extensive intramolecular and intermolecular hydrogen bonds.

In pulp and paper mills different conventional processes are used to recover cellulose from wastewater. The process of separation of cellulose from dried sludge is done using ionic liquid-based segregation techniques which involve cellulose precipitation from the sludge (Gibril et al. 2018). Direct extraction and utilization of cellulose from sawdust and woody matters are also possible.

**Fig. 12:**
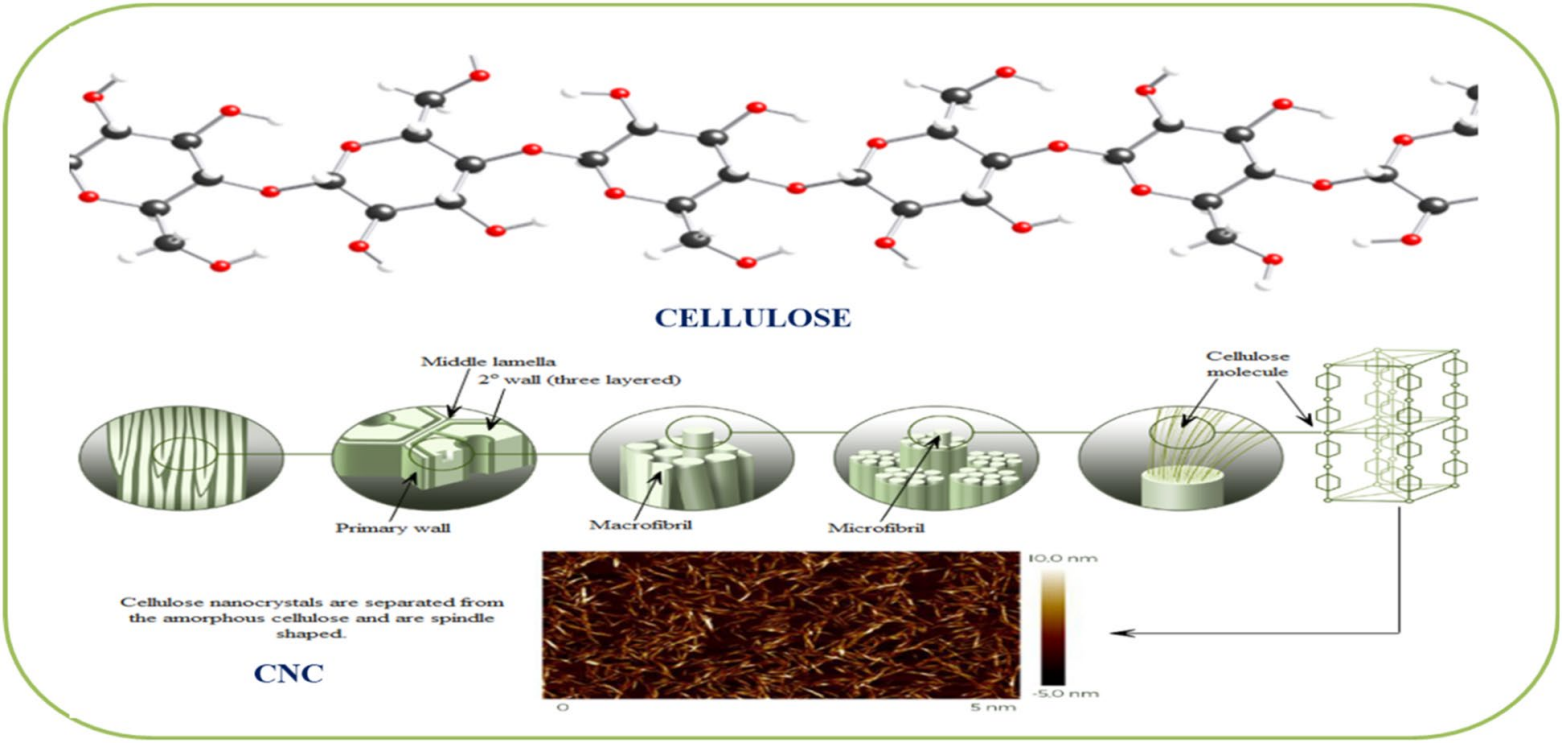
Extracting CNC from trees

The production of CNC from cellulose is an emerging possibility for the diversified utilization of trees for biomaterials (Fig. 12). A biorefinery approach can be used to convert cellulose from pulp and paper mill waste into nanocrystalline cellulose (Souza et al. 2017). The production of nanocellulose is attained by a two-step process (Clemons 2016). In the first step, the pretreatment process of native cellulose biomass is done which yields treated cellulose fibers. While in the second step, pretreated cellulose fibers are converted into nanocellulose using various routes, e.g., high-pressure homogenization, micro fluidization, micro grinding, high-intensity ultra-sonication, electrospinning, and steam explosion.

Method of synthesis of CNC generally adopts breakdown of cellulose chains to the desired nanoconfiguration via different techniques. The chemical and biological techniques utilize different chemicals and enzymes, while the physical technique involves the mechanical breakdown of the cellulose chains. The chemical process mainly involves hydrolysis using acids and alkalis or a series of reagents by the partial breaking of glucosidic bonds for obtaining the CNC. The four steps in the synthesis of CNC via mechanical method are high-pressure homogenization, fluidization to micro-level, grinding to fine particles, and finally smashing under freezing conditions. Conventionally combined methods are utilized for optimized processes and characteristics of the cellulose nanocrystal (Song et al. 2019). The biological and combined methods of the CNC synthesis method employed for hydrolysis minimize concerns with regards to environmental pollution. The production technique of CNC is well-practiced; ensuring high-grade purity of the cellulose with well-advanced extraction technology could eliminate the associated challenges in terms of high value-added products especially those used for medical items.

#### Synthesis of textile fibers from dissolving pulp

Dissolving pulp has versatile applications. Dissolving pulp can be used as a source of pulp for the paper mill (Kihlman 2012). This is particularly important as economic support for developing countries that are entirely based on imported pulp. Besides many chemicals used in washing and value additions in wet and chemical processing industries such as detergents, softeners, and binders can be produced using dissolving pulp as a precursor. These chemicals are cellulose ether-based and can be extracted from the dissolving pulp.

**Fig. 13:**
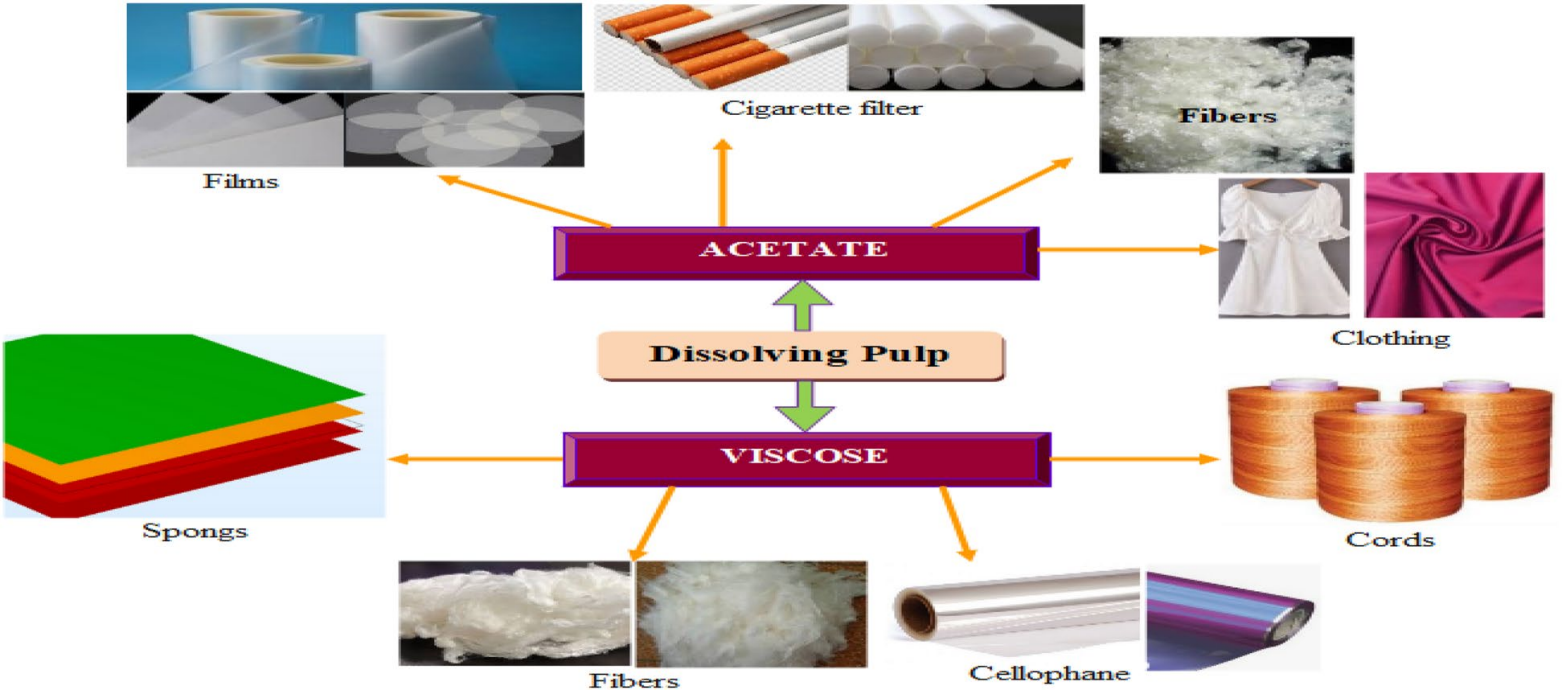
Dissolving pulp beneficiation: regenerated fibers

Dissolving pulp besides its utilization as raw material for the paper mill and surfactant synthesis it can be used for the manufacturing of textile fibers (Ma et al. 2011). The dissolving pulp can be prepared from cellulosic wastes using the same pulping route described in the current review and can be utilized for the making of viscose rayon and cellulose acetate fibers (Woodings 2001). Once the required grade of dissolving pulp is availed viscose rayon can be manufactured using wet spinning technology and cellulose acetate is manufactured using dry spinning technology. The manufactured viscose rayon and acetate fibers have diversified applications (Fig. 13).

The use of dissolving pulp as a precursor for the manufacture of commodity textile fibers has an economic impact on developing countries that entirely depend on using a single fiber such as cotton for the production of textile materials. In the region of concern, where the supporting data for availability study is conducted it has been described that the paper mill entirely depends on the imported pulp and it is not possible to produce and market the important textile fibers. It is vital to consider the economic advantage of producing potential fibers from indigenous pulp and paper wastes. Modern manufacturing techniques such as dry jet wet spinning and electrospinning techniques can be utilized for the manufacturing of different grades of fibers, especially for high-performance applications. The major challenge in the utilization of pulping waste as a precursor for textile fibers is the limited availability of fiber manufacturing units in areas, where resources are abundant; and associated yield-related problems for producing textile fibers as per the global demand.

### Biocomposites from pulp and paper mill waste

The global concerns for a safe environment and risk minimized livelihood of society are high. With this regard issues that need immediate intervention are the highest priorities of global nations. As far as feasible interventions are concerned focus needs to be given on increasing cost of petroleum and the allied depletion and frameworks in line with new environmental regulations. To this end engineering materials which have a vital impact in replacing existing petroleum-based materials and which are capable of addressing the environmental legislations are required. This was the rationale behind seeking eco-friendly green substitutes especially biocomposites for modern engineering applications (Soucy et al. 2014; Manesh 2012).

Renewable lignocellulosic materials are suitable for the reinforcement of polymers and provide biocomposites for the relevant industry. Such kinds of production trends provide relief with regards to problems encountered in using petroleum-based composites. These frequently available resources provide a remedial solution with regards to the production of attractive, sustainable, cost-effective, and eco-friendly materials so-called biocomposites with a safe environment as a result of preferred disposal and reuse options (Schorr et al. 2014).

The fly ash and other mineral-based wastes from pulp and paper mill are underutilized and fully discarded especially in developing countries (Novais et al. 2018). The fly ash is entirely composed of metal oxide minerals. Though it is not a concern of the present review the grits, dregs, and lime muds have also plenty of mineral content for specific biomaterial applications. The biomaterial which can be obtained by a suitable biorefinery technique from fly ash can be used for versatile engineering applications as a biocomposite. The prospective application of fly ash as a biomaterial is by converting the minerals into their polymer counterpart via geo-polymerization which normally are referred to as geopolymers (Mohammadkazemi 2018; Yoon-moon and Naik 2005).

Depending on the end-use the geopolymers can be incorporated in manufacturing composite materials mainly as the reinforcing component of the biocomposite. A polymer of fly ash that can be used for biocomposite is synthesized using predefined steps. The major step involves treatment with highly alkaline liquors such as using aqueous caustic soda in combination with silicate compounds such as sodium silicate in a process called alkali activation (Saeli et al. 2019; Rajamma et al. 2012). The highly alkaline environment will lead to the breaking of silica and alumina bonds in the fly ash which provides conditions for the dissolution of free silicon and aluminum ions. The reaction between the free silicon and aluminum ions with active alkali ions leads to the formation of an intermediate precursor which up on precipitation reorganize into the polymeric three-dimensional aluminosilicate structures.

Besides fly ash, sawdust from the pulp and paper mill waste is another potential source for the manufacturing of biocomposite (Zhang et al. 2020). Sawdust which mainly constitutes cellulose can be used in blending for synergistic enhancement of the reinforcement of the composite structures. The inherent biodegradability made it a preferred raw material in biocomposite applications (Zhang et al. 2020; Jiang et al. 2009).

**Fig. 14:**
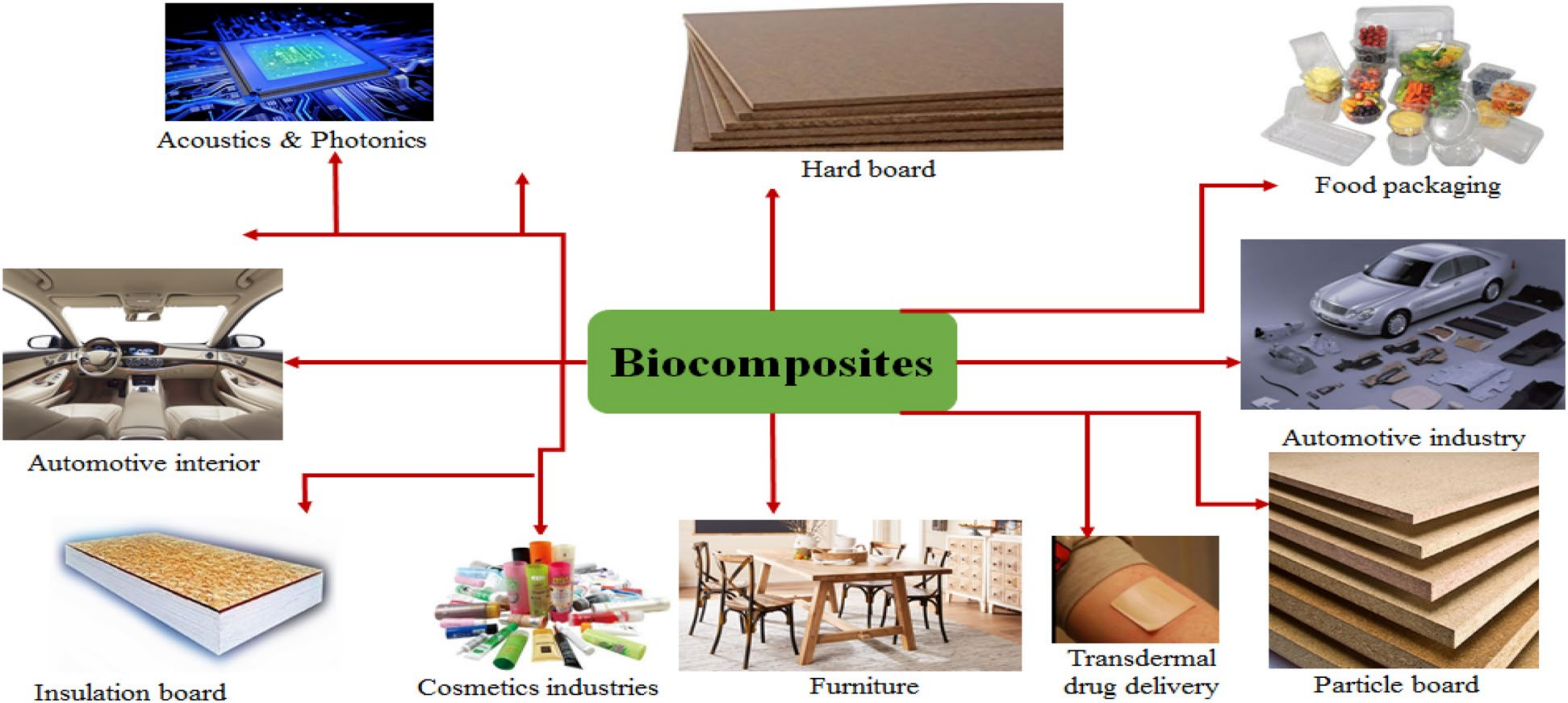
Possible applications of biocomposites from pulp and paper mill waste

Through advanced composite engineering and characterization, different biocomposites can be manufactured from sawdust and fly ash and modeled for a multitude of applications. The diverse applications of biocomposite from fly ash and saw dust-based biomaterials are summarized (Fig. 14). Both sawdust and fly ash-based biomaterial provide high heat resistant biocomposite suitable for high-performance applications. The biocomposite from sawdust cellulose can be used in the manufacturing of automotive interior, different boards, furniture, packaging, and so on, whereas fly ash based biocomposite can be used in the production of civil engineering materials mainly in construction technology (Zhang et al. 2020; Akampumuza et al. 2017; El‐Meligy et al. 2004). In both fly ash and sawdust systematic collection of sample raw material for biorefinery needs attention as they mostly are lightweight and interfere with a feasible collection. Improper management of sample collection could result in disturbance of the working environment and personal safety through inhalation needing high priority of conditioning at work stations.

## Conclusions

Pulp and paper mills release a large amount of waste globally. Most of these wastes are directly disposed to landfills with minimal incineration and nil recycling. Though there are trials for the reuse of these wastes in some of the developed world the trend is almost none in developing countries. The present review has shown that the pulp and paper mill biomass; besides their conventional use, can be converted into biomaterials that have high value for versatile applications. It is revealed that the biomass generated from the mills provides ingredients for the synthesis of biomaterials such as lignin, hemicellulose, cellulose, and various minerals. The paramount importance of biomass ingredients is that they can be converted into high value-added biomaterials using an appropriate biorefinery system. With this regard, a possibility is observed that potentially important engineering materials such as carbon fiber, bioplastic, and fibers, CNC, and biocomposites can be manufactured from waste biomass for diversified engineering applications. The synthesized biomaterials through appropriate and feasible technologies will be useful for the manufacturing of versatile bio-based products that are used in widespread conventional, high-performance, and smart applications. Future study will entail extensive research and development work to develop appropriate technologies for their full utilization and commercialization as a source of some of the proposed applications mentioned in this review. In so doing dual the impact of the newly investigated materials is realized both from the economic point of view through product diversification and environment aspects in protection and hazard minimization.

## Data Availability

All data and materials are availed in the manuscript and no additional input is required.
